# Antigen array for serological diagnosis and novel allergen identification in severe equine asthma

**DOI:** 10.1038/s41598-019-51820-7

**Published:** 2019-10-23

**Authors:** S. J. White, M. Moore-Colyer, E. Marti, D. Hannant, V. Gerber, L. Coüetil, E. A. Richard, M. Alcocer

**Affiliations:** 1 Royal Agricultural University, Cirencester, Gloucestershire GL7 6JS UK; 2 School of Biosciences, University of Nottingham, Sutton Bonington Campus, Loughborough, LE12 5RD UK; 3 Nottingham Trent University, Brackenhurst Campus, Southwell, Nottinghamshire NG25 0QF UK; 4 Department of Clinical Research and Veterinary Public Health, University of Bern, Bremgartenstr, Postfach, 3001 Bern Switzerland; 5 School of Veterinary Medicine and Science, University of Nottingham, Sutton Bonington Campus, Loughborough, LE12 5RD UK; 6 Veterinary Clinical Sciences, College of Veterinary Medicine, Purdue University, West Lafayette, IN 47907 USA; 7 LABÉO Frank Duncombe, 1 route de Rosel, 14053 Caen, Cedex 4 France; 8 Normandie Univ, UniCaen, BIOTARGEN, 3 rue Nelson Mandela, 14280 Saint-Contest, France

**Keywords:** Inflammatory diseases, Diagnostic markers

## Abstract

Severe equine asthma (sEA), which closely resembles human asthma, is a debilitating and performance-limiting allergic respiratory disorder which affects 14% of horses in the Northern Hemisphere and is associated with increased allergen-specific immunoglobulin E (IgE) against a range of environmental proteins. A comprehensive microarray platform was developed to enable the simultaneous detection of allergen-specific equine IgE in serum against a wide range of putative allergenic proteins. The microarray revealed a plethora of novel pollen, bacteria, mould and arthropod proteins significant in the aetiology of sEA. Moreover, the analyses revealed an association between sEA-affected horses and IgE antibodies specific for proteins derived from latex, which has traditionally been ubiquitous to the horse’s environment in the form of riding surfaces and race tracks. Further work is required to establish the involvement of latex proteins in sEA as a potential risk factor. This work demonstrates a novel and rapid approach to sEA diagnosis, providing a platform for tailored management and the development of allergen-specific immunotherapy.

## Introduction

Severe equine asthma (sEA) is a performance limiting, debilitating condition which is prevalent in 14% of horses in the Northern Hemisphere^1^. The pathogenesis of this condition remains controversial with many contradictory reports^2,3^; but several studies have indicated the role of immunoglobulin E (IgE) through *in vitro* histamine release assays^4–6^, and allergen-specific IgE (sIgE) analyses of bronchoalveolar lavage fluid (BALF) and sera^5,7,8^. Specific IgE assays suggest that *Aspergillus fumigatus* (*Asp f (extract), rAsp f 8*, Asp f 1/a)*, Alternaria alternate*, *Tyrophagus putrescentiae*, *Saccharopolyspora rectivirgula, Aspergillus terreus, Eurotium amstelodami, Geotrichum candidum* and *Wallemia sebi* may be implicated in the aetiology of sEA^2,3,8–11^. More recently, White *et al*., (2017) identified 40 potential allergens of interest, from several genera, including fungi, bacteria, pollen and arthropod^12^.

sEA diagnosis is presently conducted on clinical history and readily identified clinical signs^13^, which have been shown to correlate with sEA severity^14^, with ancillary diagnostic tests such as BALF cytology, lung function testing, haematology, and immunological testing used to improve diagnostic accuracy^15^. While several studies have addressed the potential benefits derived from *in vitro* allergen assessment in diagnosis of sEA, commercial application has been hampered due to a lack of statistical approaches for clear disease classification, and the limited range of allergens tested to date.

More recently, White *et al*., (2019) developed microarray methods to enable IgE profiling in sEA-affected horses, elucidating previously unidentified causal allergens and demonstrating a strong correlation between BALF and sera specific IgE profiles^16^. The aim of the present study was to use sera from a large group of horses from France, Switzerland, USA and Canada, exposed to a wide range of potential allergens in the normal equine environment and determine if a combination of microarray and mathematical modelling could be used to elucidate previously unidentified allergens involved in the aetiology of sEA, as a potential diagnostic test for sEA, and to evaluate the influence of samples from mixed environments without matched controls. To achieve this, we primarily used specific allergen molecules, thus identifying genuine sensitisation and minimising cross-reactivity, potentially enabling precise allergen selection for future immunotherapy.

## Materials and Methods

### Equine sera samples

Horses from Canada, France and the US were classified according to clinical assessment, including physical examination, tracheal mucus, pulmonary function test, reversible airway obstruction after medical/environmental change and BALF cytology, demonstrating moderate to severe neutrophilia (>25% cells), as previously described^15^. Control horses had no record of lung disease, no previous history of laboured breathing, coughing or nasal discharge, no tracheal mucus, and <10% BALF neutrophils. Swiss samples were those published in Verdon *et al*.^17^, sEA was classified using the horse owner assessed respiratory signs index (HOARSI) ≥3 and partial pressure of arterial oxygen <90 mm Hg, and Insect Bite Hypersensitivity (IBH) classified via IBH scoring^17^. Blood was collected from the jugular vein in VACUETTE Serum Clot Activator Tubes, centrifuged at 2000 × g for 10 minutes, serum removed and stored at −80 °C. This study was approved by the Royal Agricultural University Ethical Review Group. All experiments were performed in accordance with the relevant guidelines and regulations.

In the first part of the study, a sub-group of the total of 138 sports horses, consisting of n = 35 environmentally matched samples from France (5 sEA; 6 control), USA (6 sEA; 6 control) and Canada (6 sEA; 6 control) were analysed. These were modelled to enable reliable comparison of samples with matched controls collected from horses in the same environment, thus accounting for any antigenic stimuli associated IgE responses. In order to test the robustness and clinical relevance of the test, in the second phase of the study, microarray analysis was carried out on a larger group, including the aforementioned horses and those from differing environments without matched controls (n = 138), consisting of sEA n = 33, IBH/sEA n = 23, IBH n = 24 and control n = 58 from France, Switzerland, USA and Canada. Horses suffering with IBH, a classic equine hypersensitivity, were included to further assess the discriminatory power and clinical relevance of this approach. This group (n = 138) was used to build and test the mathematical predictive model and identify relevant allergens.

### IgE sera determination by protein microarray

The comprehensive complex microarray comprised of extracts (n = 153) and pure proteins (n = 231) from a wide range of fungi, bacteria, pollen, arthropods and others associated with the equine environment. The extracts and pure proteins were obtained from commercial suppliers, produced in-house and donated to our group. Fungi and bacteria strains were purchased from Deutsche Sammlung von Mikroorganismen und Zellkulturen, grown in liquid media, and extracts produced via sonication. Samples were normalised to 0.5 mg/ml protein and printed onto ONCYTE® NOVA Nitrocellulose Film Slides (Grace Bio-Labs, Oregon, USA) using an Ultra Marathon II by Arrayjet, (Roslin, Scotland) to a final spot density of 12,288 spots/slide, with an approximate spot size of 200 μm diameter and replicated twice into two blocks on each pad. For alignment purposes Cy3/Cy5 were included, and for quality control purposes a number of sham antigens were spotted (e.g. PBS, Equ c 3, Ara h 1-NT, Man e, Gal d 1–4). Slides were blocked in 3% BSA (w/v) in PBS inside a Corning 5 slide holder (product # 40082) using a mini hybridization oven (Appligene, USA) at 37 °C for 3 h, washed three times for 2 min in PBS containing 0.05% (w/v) Tween-20, followed by five times 1 min washes with Milli-Q water, and dried via centrifugation (MSE Mistral 3000i, Sanyo, UK) 300 × g for 10 min at room temperature.

Slides were fitted with Proplate slide modules (Grace Bio-Labs, product # 204862) and washed three times (60 second dwell time) with PBST (0.2%). Sera samples were diluted 1:2 with 4% BSA in 0.4% PBST, and 100 μl of prepared sample was added to each well, excluding well 4, which was a control filled with 100 μl of the dilution solution (1:2) in 2% BSA in 0.2% PBST (final dilution). The Proplate was fitted with an adhesive seal strip and incubated for 16 hours at 4 °C on the Stuart mini see-saw rocker (SSM4) at 13 oscillations/minute. Slides were washed three times with PBST (0.05%) using the BioTek plate washer and incubated for 2 hours at 37 °C in a ThermoHybaid (HyPro 20) at AVS 3 with 100 μl per well of anti-horse IgE (BioRad, #MCA5982GA) 1:400 in 1% BSA in 0.2% PBST. They were washed a further 3 times with PBST (0.05%) and incubated for one hour at 37 °C in the ThermoHybaid with 100 μl per well of DyLight 649 conjugated anti-mouse IgG (Rockland, Product #610-443-040) 1:400 in 1% BSA in 0.2% PBST. Slides were then washed three times with PBST (0.05%) followed by three washes with Milli-Q water and dried via centrifugation at 300 × g for 10 mins (Mistral 3000i, rotor 43124-708).

### Data analysis

Processed slides were scanned in a GenePix 4000B (Molecular Devices, USA) with the PMT settings 440 and 310 at 635 and 532 nm and saved as TIF files. Images were processed in GenePix Pro software v6.0.1.27 (Axon Instruments) and saved as comma-delimited text files. Digital fluorescence units (DFUs) were calculated for each spot by subtracting local background from the median fluorescence value of the spot. One pad on each microarray was used as a control, containing reagents and no serum, the results of which were subtracted from all other pads to account for any auto-fluorescence or non-specific binding. Clinically healthy and IBH horses were used as control.

PLS toolbox (version 5.8.3, Eigenvector Research Inc., USA) running on a MATLAB platform (MathWorks, Cambridge, UK) was used to carry out principal component analysis and partial least squares discriminant analysis (PLS-DA) was used as a classifier which enabled construction of the predictive mathematical models^7^. Partial Least Squares Discriminant Analysis (PLS-DA), a type of PLS regression against a dummy matrix, was used to separate pre-defined classes of samples (i.e. affected/non-affected horses). The model was used to inform which specific variables (allergens) are important to determine class prediction^18^. A variable influence on the projection (VIP) score of each variable was calculated as a weighted sum of the squared correlations between the original variable and the PLS-DA components. This is a measure of the contribution that a specific variable has on the model^19^. In order to test the mathematical model produced, multiple rounds of cross validation (CV) were performed using different partitions, and the validation results were amalgamated through the rounds giving an estimate of the model’s predictive performance^19^.

## Results

### Environmentally matched group

**Figure 1:**
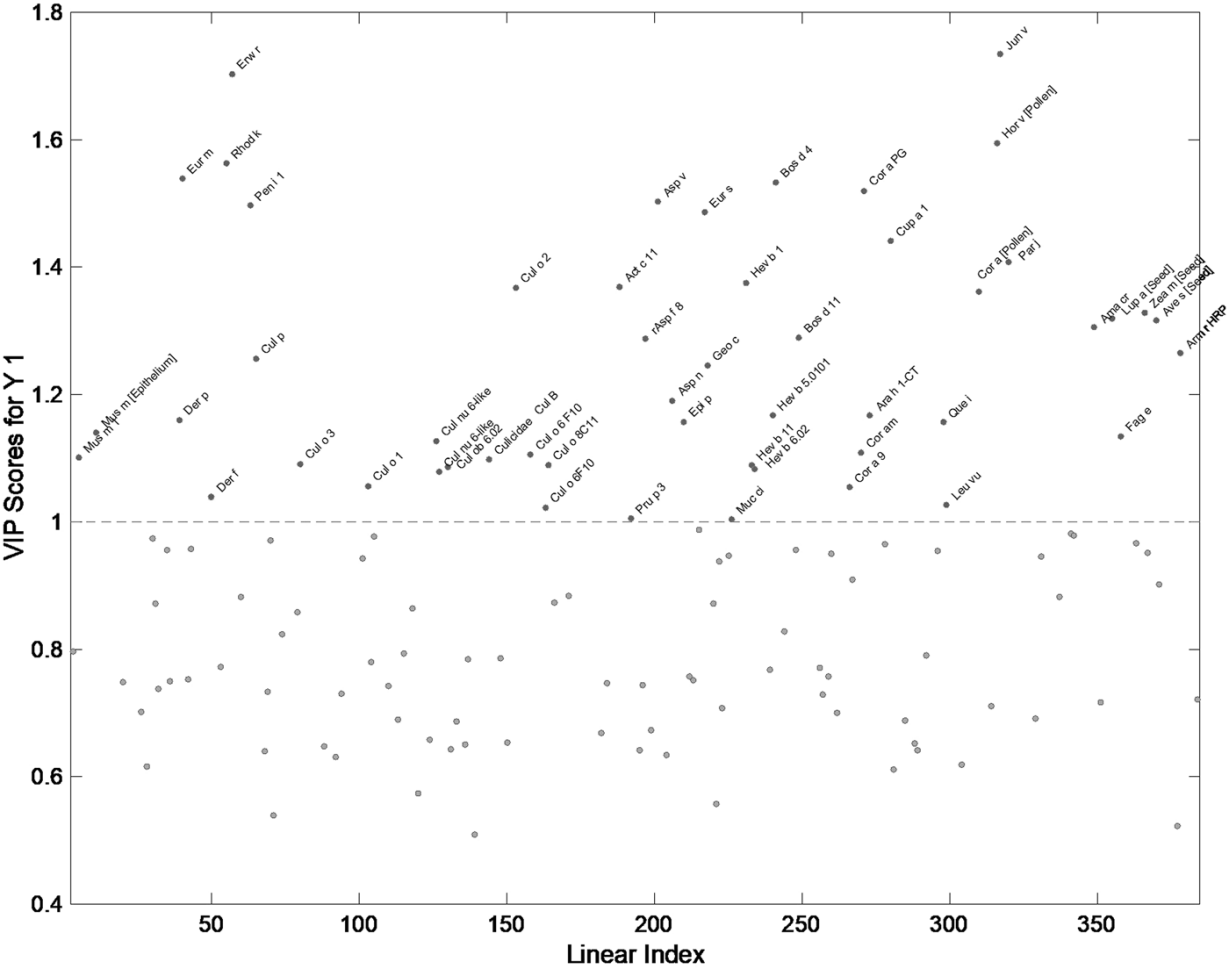
Variable influences on the projection calculated by PLS-DA software from the environmentally matched group of horses (n = 35) after VIP selection. A threshold of α > 1 was used to identify those VIPs significant in class prediction.

The initial calibration of the PLS-DA classification method using the small subset of environmentally matched samples (n = 35) was highly encouraging, with CV values confirming good prediction (Table 1). In an effort to reduce the background noise and improve robustness of the mathematical model, a second round of modelling was conducted using the main VIPs (n = 129) identified in the calibration step (Fig. 1). This improved both sensitivity and specificity of the (CV) mathematical model (Table 1).

**Table 1:** Partial least squares discriminant analysis statistics of the calibrated and cross validated data from the environmentally matched group of horses (n = 35) from the first (before VIP selection) and second (after VIP selection) rounds of modelling.

	Before VIP selection		After VIP selection	
	CAL	CV	CAL	CV
Specificity	1.00	0.70	1.00	1.00
Sensitivity Error	1.00 RMSEC = 0.092	0.72 RMSECV = 0.480	1.00 RMSEC = 0.052	0.94 RMSECV = 0.275

CAL = calibration; CV = cross validation.

### Environmentally mixed group

The mathematical calibration model with the reduced number of sEA horses and matched samples has shown that it is feasible to discriminate sEA from control animals. Whether other allergic diseases and samples without matched controls would interfere with the detection and classification method has been tested using a cohort of 138 horses (34 sEA, 23 IBH/EA, 23 IBH and 58 controls). The PLS-DA calibration modelling involving this new cohort (n = 138) confirmed the good prediction for sEA obtained with non-matched samples, particularly after the second round of mathematical modelling using the sEA VIP selection (Table 2). A wide range of VIPs were identified as significant for class prediction (see Supplementary Data), predominant variables included Hev b 11, Hev b 6.02, Hev b 5.0101, rAsp f 8 and Hel as 7.

**Table 2:** Partial least squares discriminant analysis statistics of the calibrated and cross validated data from the environmentally mixed group of horses (n = 138) showing different classification values after sEA VIP selection.

	sEA		Control		IBH	
	CAL	CV	CAL	CV	CAL	CV
Specificity	0.865	0.865	0.787	0.725	0.957	0.902
Sensitivity	0.765	0.735	0.931	0.81	0.826	0.674
Error	RMSEC = 0.315	RMSECV = 0.360	RMSEC = 0.366	RMSECV = 0.413	RMSEC = 0.108	RMSECV = 0.366

CAL = calibration; CV = cross validation.

## Discussion

Previously, using Partial Least Squares Regression we demonstrated that human IgE microarray analysis with extracts and pure proteins correlate well with standard laboratory methods such as ELISA, UniCAP and immunoblot test^20^. Similarly, there was good agreement between equine IgE microarray and ELISA results in this study (see Supplementary Data). Mathmatical modelling of profiling data for disease classification and allergen identification is well utilised in human alogology, but has had little application in the veterinary sector^7^. Based on these principles, we utilised latest technological developments and mathematical modelling to explore sEA. This enabled the widest scale sEA-associated allergen profiling to date. This study utilised whole protein extracts to maximise allergen coverage, while maintaining specificity by including purified proteins where allergens were known. This was essential because of the limited numbers of potential sEA allergens screened by others to date. Further work would benefit from purifying proteins of the identified whole extracts of interest, thus providing well-defined reagents for component resolved diagnostics enabling increased specificity and sensitivity, particularly aimed at the use of specific immunotherapy^21^.

Many of the allergens identified in the initial model (Fig. 1) have previously been implicated in human allergic asthma, but not previously assessed in the horse. *Erwinia, Geotrichum candidum* and *Eurotium amstelodami* have been associated with asthma and occupational respiratory diseases in farmers^9,22–24^. Moreover, *Junioerus virginiana* and *Corylus avellane* pollen are often noted as inciting hay fever and asthma^25^. Particularly noteworthy allergens include *Aspergillus versicolor*, *A niger* and *A fumigatus* all from the most significant genus associated with the aetiology of human asthma and sEA^24^. Similarly, *Dermatophagoides farinae* is associated with human asthma as well as sEA^11,26^, and Pen i 1 is cross-reactive to many arthropods^27^. Furthermore, several latex allergens are significant for class prediction, a group previously untested in the horse.

**Figure 2:**
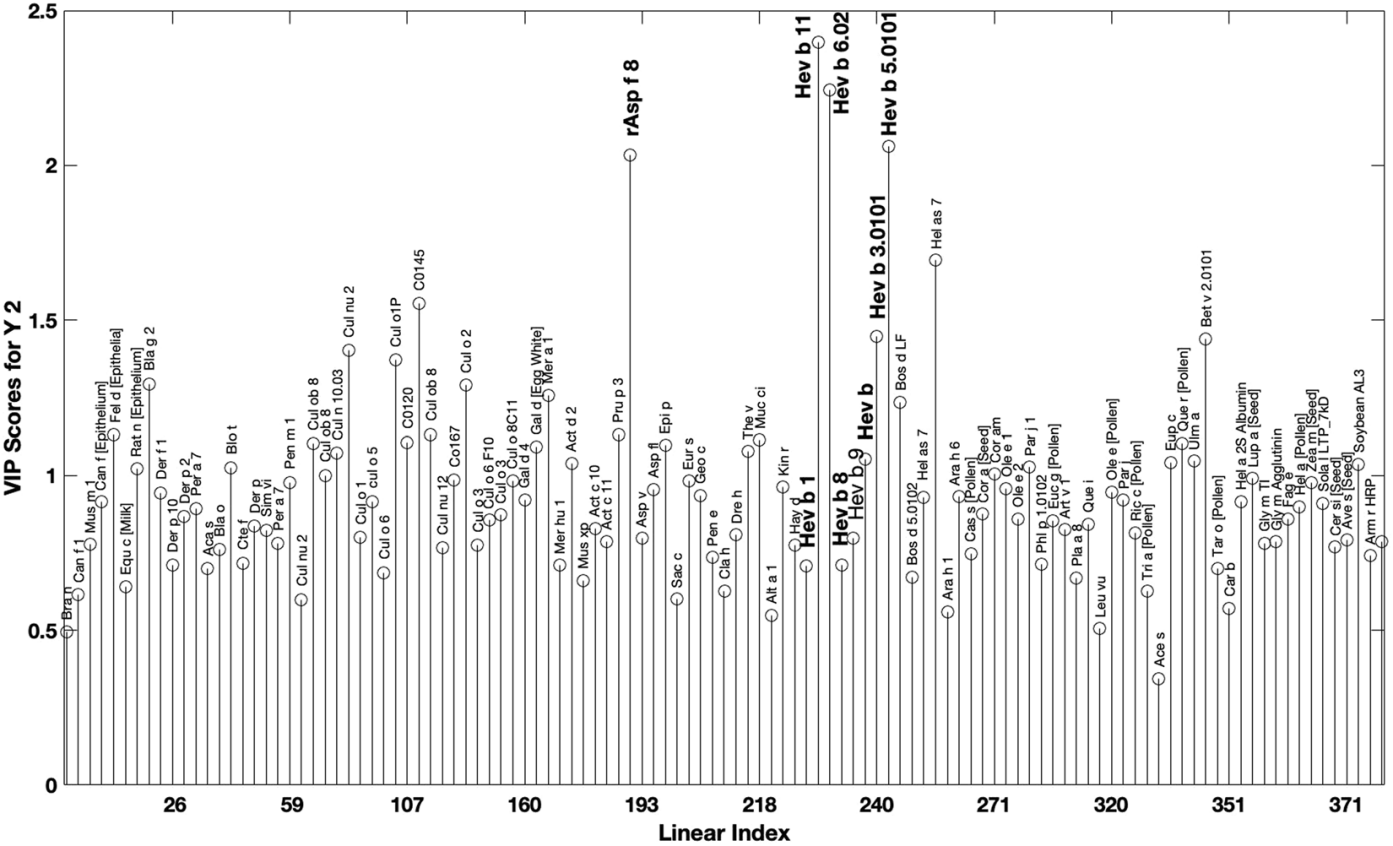
Variable influences on the projection (VIP) scores calculated by PLS-DA from the environmentally mixed group of horses (n = 138) after VIP selection. A threshold of α > 1 was used to identify those VIPs significant in class prediction.

The classification results (CV sensitivity and specificity) from the second model (Fig. 2) were however lower than the first small subset of horses. The discrepancies between the two subgroups most likely results from the second group being collected from varying environments as described by Eder *et al*.^8^. These authors assessed 450 horses from 6 different yards and concluded stable-specific environments have a highly significant effect on allergen-specific serum IgE levels. The second group presented here did not have matched controls to account for environmental-associated IgE production and possessed a strong IBH response bias which may have weakened the mathematical predictive model. The most influential VIPs for class separation were those from natural rubber latex (Hevea brasiliensis, Hev b), these included Hev b 11; Hev b 6.02; Hev b 5.0101; Hev b 3.0101 and Hev b-extract (see Supplementary Data). To the authors knowledge, this is the first time Hev b allergens have been assessed in relation to sEA. As shown in Fig. 2, a smaller level of IgE-binding to latex allergens was detected in the sera of IBH positive horses and controls used in this study (latex means: 1445, 735, 803 for sEA, IBH and control respectively with P < 0.0001 when compared to sEA), however latex allergens alone were not able to discriminate the sEA group. As shown in Table 2 this discrimination is much improved with the other VIPs, particularly *Aspergillus* (Asp f 8).

Work in human asthma patients has revealed a higher frequency of Hev b allergies in affected individuals^28^. A major source of respirable Hev b allergens in the horse’s environment is from artificial riding surfaces. Although the use of recycled tyres was banned in many parts of Europe in 2007, in the UK it is permitted under current Environment Agency waste regulations (Waste Exemption: U8 use of waste for a specified purpose), and many arena surfaces throughout the world contain components of natural rubber. These surfaces have high levels of respirable dust, which has previously been associated with chronic bronchitis in riding instructors^29–31^. Respiration of Hev b particles have also been shown to induce inflammation and oxidative stress in the lungs of humans^32^. Furthermore, particles, such as Hev b, have been shown to exhibit an adjuvant effect by increasing the primary response during sensitisation when present either before, during or after allergen exposure^33^. Diaz-Sanchez *et al*., (1999) demonstrated particulate inhalation during allergen exposure could induce a mucosal IgE response under conditions in which the allergen alone could not^34^. Similarly, experimental animal models in strains of mice not prone to developing IgE responses, demonstrated that particulate antigens may enhance sensitisation^35^. Given the adjuvant and sensitising effects of latex, these airborne particles could contribute to the increase in both latex sensitisation and asthma through direct and indirect mechanisms^36,37^, which may explain the association between sEA and Hev b-specific IgE demonstrated here. Moreover, these results are in agreement with previous work identifying the urban environment, which is high in respirable natural rubber latex^37^, as a risk factor in sEA^38^. In humans, regular exposure to latex particles in the work environment can lead to occupational asthma, commonly known as latex-induced asthma. The prevalence of latex sensitisation in occupationally-unexposed groups is significantly lower (<1%) than those regularly exposed (>18%)^39^. The main allergen associated with occupational latex-allergy (Hev b 6.02)^40^ was the second most influential VIP in our study group with sEA-affected horses, along with other major Hev b-allergens used for occupational latex-allergy diagnosis (Hev b 11; Hev b 5.0101)^41^. The results of this study would suggest there may be an association between sEA and increased latex-specific IgE. Further equine specific work is required to establish the exposure levels of latex in the horse’s daily environment, demonstrating the benefit of latex avoidance, latex inhalation reactivity tests, epidemiological studies and further hypersensitivity confirmation through basophil activation tests. At present, exposure should be considered a potential risk to the respiratory health of the horse.

Several fungal allergens were found to significantly influence class prediction, these included *Aspergillus fumigatus* (rAsp f 8), *Mucor circinelloides f. lusitanicus* (Muc ci), *Geotrichum candidum* (Geo c) and *Eurotium amstelodami* (Eur a) (see Supplementary Data). The rAsp f 8 results confirm those of Eder *et al*., (2000) and Künzle *et al*., (2007) whom also found significantly more IgE against this recombinant mould allergen in sEA-affected horses^8,10^. Tahon *et al*., (2009) also reported significantly higher positive intradermal reactions to rAsp f 8 in sEA-affected horses^42^. *Mucor circinelloides f. lusitanicus* (Muc ci) results further confirm previous research demonstrating *Mucor* allergen extract sensitisation is associated with sEA-affected horses via *in vitro* basophil assay^5^. Similarly, increased levels of specific IgE against *E. amstelodami* and *G. candidum* have been identified in the bronchoalveolar lavage fluid of sEA affected horses via western blot^9^. Several arthropods were significant for class separation, including the tropomyosin’s of *Helix aspersa* (Hel as 7 and *Periplaneta Americana* (Per a 7), the proteases from *Blattella germanica* (Bla g 2) and *Dermatophagoides farinae* (Der f 1), the complex mixture of *Blomia Tropicalis* (Blo t), and *Dermatophagoides pteronyssinus* (Der p 2). The array results therefore ratify recent reports on the involvement of *Acarus siro*, *Dermatophagoides, farinae/pteronyssinus, Tyrophagus putrescentia* in sEA and their association with high concentrations of specific IgE against mites, particularly *T putrescentia*^11^. Bla g 2 is associated with the development of asthma in humans and increased sIgE against Bla g has previously been reported in sEA-affected horses^16,43^. Tropomyosin results (Hel as 1 and Per a 7) are to be expected, as Tropomyosin are major allergenic components accounting for cross-reactivity with mites and other arthropods^44^. Furthermore, the high VIP scores demonstrated for *Cullicoides* proteins (Cul nu 2, CO145, Cul o 2) could have resulted from the sEA/IBH horses, even though these were matched with IBH controls, or from multiple hypersensitivities, as sEA horses are at increased risk of IBH^45^ which is associated with airway hyperreactivity^46^. The only bacteria considered significant for class separation was *Thermoactinomyces vulgaris*, which has long been associated with sEA and increased levels of IgE in affected horses^47–49^. Interestingly, our study showed 28 pollens were significant for class separation, including *Betula verrucosa* (Bet v 2.0101), *Mercurialis annua* (Mer a 1), *Eupatorium capillifolium* (Eup c), *Quercus robur* (Que r) and *Helianthus annuus* (Hel a). To the authors knowledge, this is the first study to show an association between sEA in horses and a hypersensitivity to pollens. When utilising a panel of 131 allergens, Einhorn *et al*., (2018) demonstrated that horses are most likely to be sensitised to Fag e 2, Cyn d 1 and Aln g 1, similarly here we found Fag e was significant for class prediction (Fig. 2)^50^.

As expected, the environmentally matched (MA) group has several VIPs in common with the environmentally mixed (MI) group. Most notably Hev b 11, Hev b 6.02, rAsp f 8, Eur s and Hev b 5.0101. Moreover, many similarities are apparent, such as Der f and tropomyosin Pen i 1 in MA compared with Der f 1 and tropomyosin Hel as 7 in MA. The MI group was equally reliant on a range of aspergillus species (Asp v, Asp n, rAsp f 8), whereas the MA group primarily relied on rAsp f 8. Bovine milk proteins are important for class prediction in both models (MA - Bos d 4, Bos d 9; MI - Bos d LF), the significance of this warrants further research, these molecules commonly cross react between species and have shared common allergenic components with other allergens, such as Glycine max^51^.

Use of a PLS-DA model enabled the classification of sEA-affected horses using IgE as a biomarker, which has previously not been possible with the utilised statistical methods due to overlap between affected and non-affected groups^8,50^. Such models have been employed in the human sector to enable diagnosis of asthma patients using metabolomics with great success, and proved to be just as effective with sEA^2,7,19^. Furthermore, the identification of specific IgE auto-reactivity through VIP identification contributes to an understanding of the pathogenesis of the disease. The ability to discriminate sEA-affected horses from other IgE-mediated conditions demonstrates the robustness of the test. Further research expanding the repertoire of allergens tested in the form of pure proteins would increase the diagnostic accuracy of the mathematical model as well as benefiting identification of genuine sensitisation and enabling therapeutic and diagnostic development. This advanced bioinformatics enabled the largest scale allergen profiling of sEA to date, significantly contributing to aetiological understanding of this complex disease.

In conclusion, the microarray platform demonstrated here may be utilised as axillary diagnosis for sEA, informing accurate allergen-avoidance regimes based on its sensitisation profiles; while simultaneously elucidating important factors associated with the aetiology and pathogenesis of this complex disease. Moreover, it enables further diagnostic developments and the creation of specific immunotherapy treatments. This serological investigation of 138 horses living in varying environments identified that sEA is associated with a large sensitisation profile, and predominantly involves latex, fungi, mite and pollen proteins; demonstrating similar profiles to that found with allergic asthma in the human. These results indicate that exposure to latex may be detrimental to the respiratory health of the horse. Further research is required to establish the levels of latex exposure in the equine environment and its *in vivo* effects. Sensitivity and specificity values confirmed the high discriminatory power of the technique in combination with mathematical modelling. The microarray platform demonstrated here will enhance the health, welfare and performance of sEA affected horses. This has been achieved on a number of levels through (a) the development of a novel serological diagnostic test, (b) improved understanding of disease pathogenesis, and (c) identification of novel allergenic candidates.

## Supplementary information

Supplementary data

## Data Availability

The datasets generated and/or analysed during the current study are available from the corresponding author on reasonable request.
