# Supplementary material for: Antigen array for serological diagnosis and novel allergen identification in severe equine asthma
Source: Sci Rep. 2019 Oct 23;9:15170. doi: 10.1038/s41598-019-51820-7 (PMC6811683; doi:10.1038/s41598-019-51820-7)
Supplement: Supplementary file 1 — Supplementary data [file 41598_2019_51820_MOESM1_ESM.pdf]

Antigen array for serological diagnosis and novel allergen identification in severe equine asthma

S. White[1, 2, 3*], M. Moore-Colyer[1], E. Marti[4], D. Hannant[5], V. Gerber[4], L. Coüetil[6], E.A. Richard[7, 8] and M. Alcocer[2].

[1]Royal Agricultural University, Cirencester, Gloucestershire, GL7 6JS, UK.

[2]School of Biosciences, University of Nottingham, Sutton Bonington Campus, Loughborough, LE12 5RD, UK.

[3] Nottingham Trent University, Brackenhurst Campus, Southwell, Nottinghamshire, NG25 0QF, UK.

[4]Department of Clinical Research and Veterinary Public Health, University of Bern, Bremgartenstr, Postfach, 3001 Bern, Switzerland.

[5]School of Veterinary Medicine and Science, University of Nottingham, Sutton Bonington Campus, Loughborough, LE12 5RD, UK.

[6]Veterinary Clinical Sciences, College of Veterinary Medicine, Purdue University, West Lafayette, IN, 47907, USA.

[7]LABÉO Frank Duncombe, 1 route de Rosel, 14053 Caen Cedex 4, France.

[8]Normandie Univ, UniCaen, BIOTARGEN, 3 rue Nelson Mandela, 14280 Saint-Contest, France.

*samuel.white@ntu.ac.uk

Bland Altman plots to compare ELISA and microarray IgE with specific proteins. The acceptable level of bias is dictated by the solid line. The dashed lines represent the 95% limits of agreement as the mean difference (2 SD).

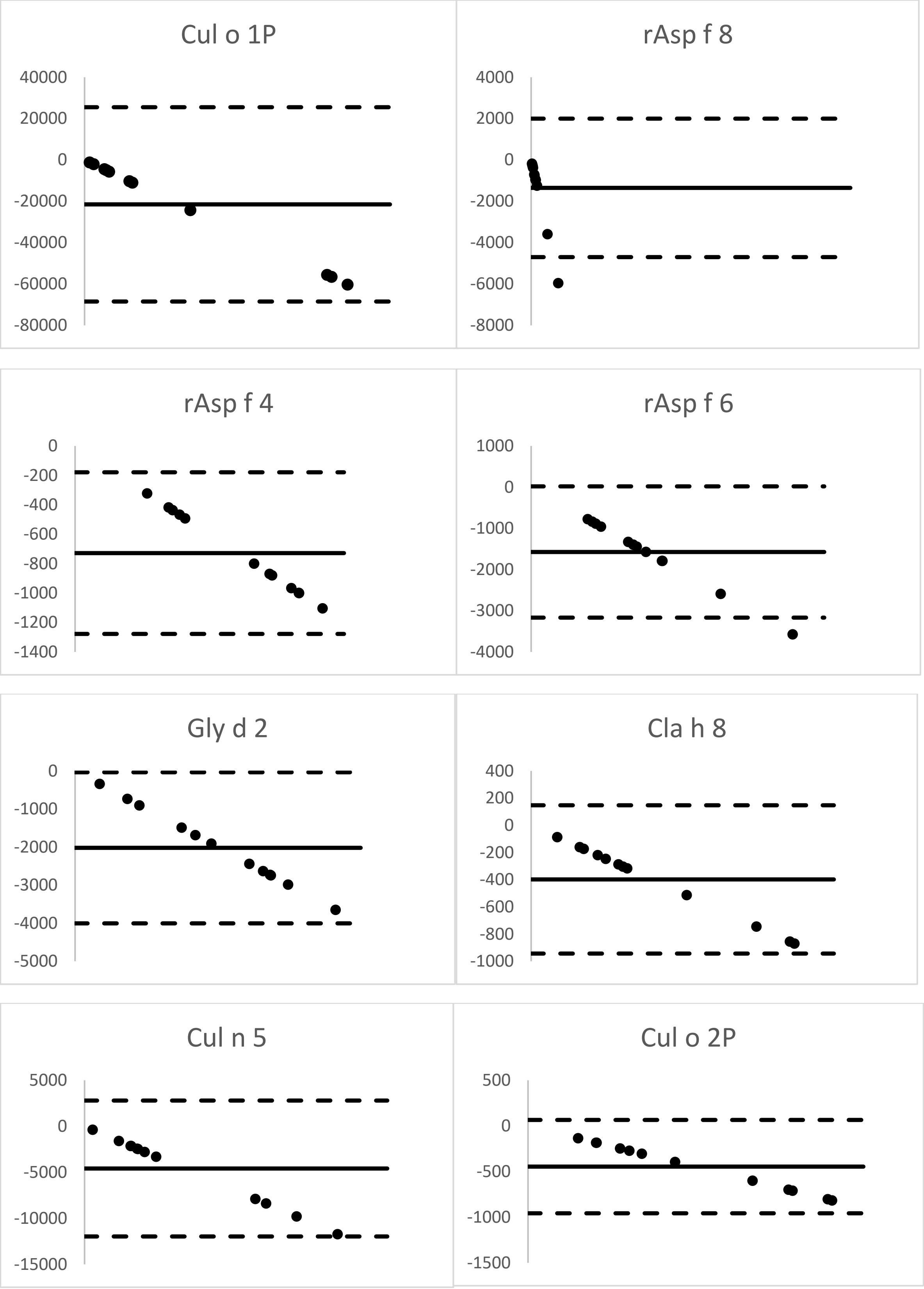

Summary of horses used in the study.

	**Switzerland**	**France**	**USA**	**Canada**
Number of horses	16 sEA affected<br><br>24 IBH affected<br><br>23 IBH/sEA affected<br><br>40 control	5 sEA affected<br><br>6 control	6 sEA affected<br><br>6 control	6 sEA affected<br><br>6 control
Horse Owner Assessed Respiratory Signs Index (HOARSI) (Ramseyer et al., 2007)	†	na	na	na
Histamine/Metacholin Bronchoprovocation : (concentration of histamine when inhalation was stopped)	†	na	na	na
Partial pressure of arterial oxygen	†	na	na	na
Provocation concentration	†	na	na	na
Pulmonary function test	†	†	†	†
Insect bite hypersensitivity score (Etude, 2014)	†	na	na	na
Physical examination : including respiratory and cardiac auscultation, rectal temperature, capillary refill time and colour of mucous membranes, palpation of submandibular lymph nodes, lung auscultation at rest and during rebreathing	†	†	†	†
Mucus scoring system	na	†	†	†
Bronchoalveolar lavage fluid (including cytology)	na	†	†	†
Owner reported coughing, nasal discharge at time of examination	†	†	†	†
Reversible airway obstruction (after medical/environmental change)	na	†	na	na

† : measured in this group
na : not analysed

Variable influences on the projection scores significant for class prediction from the environmentally mixed group of horses (n=138) after VIP selection.

**Species**	**Allergome name**	**Extract = E; Molecule = M**	**VIP score**
Hevea brasiliensis	Hev b 11	M	2.397886012
Hevea brasiliensis	Hev b 6.02	M	2.242175574
Hevea brasiliensis	Hev b 5.0101	M	2.061708356
Aspergillus fumigatus	rAsp f 8	M	2.034462948
Helix aspersa	Hel as 7	M	1.693216892
Culicoides obsoletus	Cul o 145	M	1.553348148
Hevea brasiliensis	Hev b 3.0101	M	1.447433432
Betula verrucosa	Bet v 2.0101	M	1.439197666
Culicoides obsoletus	Cul nu 2	M	1.401734766
Culicoides nubeculosus	Cul o1P	M	1.370674276
Blattella germanica	Bla g 2	M	1.294109254
Culicoides obsoletus	Cul o 2	M	1.289809746
Mercurialis annua	Mer a 1	M	1.257180767
Bos domesticus	Bos d LF	M	1.234823184
Culicoides obsoletus	Cul ob 8	M	1.132147319
Prunus persica	Pru p 3	M	1.131899686
Felis domesticus	Fel d [Epithelia]	E	1.131671083
Mucor circinelloides	Muc ci	E	1.113604072
Culicoides obsoletus	C0120	M	1.107017518
Quercus robur	Que r [Pollen]	E	1.103588161
Culicoides obsoletus	Cul ob 8	M	1.10169779
Epicoccum nigrum	Epi p	E	1.097080342
Gallus domesticus	Gal d [Egg White]	E	1.092020409
Thermoactinomyces vulgaris	The v	E	1.078503603
Culicoides obsoletus	Cul n 10.03	M	1.070733631
Hevea brasiliensis	Hev b	E	1.053235133
Ulmus americana	Ulm a	E	1.046124579
Eupatorium capillifolium	Eup c	E	1.041304179
Actinidia deliciosa	Act d 2	M	1.039211209
Glycine max	Soybean AL3-recombinant	M	1.036586734
Parietaria judaica	Par j 1	M	1.025947792
Blomia tropicalis	Blo t	E	1.024506004
Rattus norvegicus	Rat n [Epithelium]	E	1.022811408
Corylus americana	Cor am	E	1.00432009
Culicoides nubeculosus	Cul ob 8	M	0.999496997
Lupinus albus	Lup a [Seed]	E	0.990564278
Culicoides obsoletus	Co167	M	0.983835139

Culicoides obsoletus	Cul o 8C11	M	0.98257687
Eurotium amstelodami	Eur s	E	0.981731287
Penaeus monodon	Pen m 1	M	0.977846295
Zea mays	Zea m [Seed]	E	0.975934894
Kineosporia rhizophila	Kin r	E	0.96268091
Olea europaea	Ole e 1	M	0.957886893
Aspegillus flavus	Asp fl	E	0.954692807
Olea europaea	Ole e [Pollen]	E	0.946553558
Dermatophagoides farinae	Der f 1	M	0.943956016
Geotrichum candidum	Geo c	E	0.934810722
Arachis hypogaea	Ara h 6	M	0.931278284
Helix aspersa	Hel as 7	M	0.929469269
Gallus domesticus	Gal d 4	M	0.92158607
Parietaria judaica	Par j	E	0.919641275
Culicoides	cul o 5	M	0.915549349
Helianthus annuus	Hel a 2S Albumin	M	0.915519266
Canis familiaris	Can f [Epithelium]	E	0.914611185
Solanum lycopersicum	Sola l LTP_7kD	M	0.909716836
Helianthus annuus	Hel a [Pollen]	E	0.898832645
Periplaneta americana	Per a 7	M	0.892563113
Corylus avellana	Cor a [Seed]	E	0.877063468
Culicoides obsoletus	Cul o 3	M	0.87297805
Dermatophagoides pteronyssinus	Der p 2	M	0.866653724
Olea europaea	Ole e 2	M	0.859525551
Fagopyrum esculentum	Fag e	E	0.859249296
Culicoides obsoletus	Cul o 6 F10	M	0.856052813
Eucalyptus globulus	Euc g [Pollen]	E	0.854059354
Quercus ilex	Que i	E	0.842572502
Dermatophagoides pteronyssinus	Der p	E	0.836111501
Actinidia chinensis	Act c 10	M	0.829294987
Artemisia vulgaris	Art v 1	M	0.824564544
Black fly	Sim vi	E	0.823110421
Ricinus communis	Ric c [Pollen]	E	0.815160266
Drechslera halodes	Dre h	E	0.807378063
Culicoides nubeculosus	Cul o 1	M	0.799266712
Hevea brasiliensis	Hev b 9	M	0.796595977
Aspergillus versicolor	Asp v	E	0.796427599
Avena sativa	Ave s [Seed]	E	0.790808149
Actinidia chinensis	Act c 11	M	0.787068315
Manioc	Man e	E	0.785608616
Glycine max	Gly m Agglutinin	M	0.78479311
Glycine max	Gly m TI	M	0.781228253

Periplaneta americana	Per a 7 (0.5mg)	M	0.779612014
Mus musculus	Mus m 1	M	0.777665393
Culicoides obsoletus	Cul o 3	M	0.776157103
hay dust	Hay d	E	0.774529898
Ceratonia siliqua	Cer si [Seed]	E	0.767985414
Culicoides nubeculosus	Cul nu 12	M	0.765522432
Blatta orientalis	Bla o	E	0.760381536
Castanea sativa	Cas s [Pollen]	E	0.748034147
Armoracia rusticana	Arm r HRP	E	0.740446527
Penicillium expansum	Pen e	E	0.734560812
Ctenocephalides felis	Cte f	E	0.716428089
Phleum pratense	Phl p 1.0102	M	0.712552232
Hevea brasiliensis	Hev b 8	M	0.710739268
Merluccius hubbsi	Mer hu 1	M	0.71046373
Dermatophagoides pteronyssinus	Der p 10	M	0.7096095
Hevea brasiliensis	Hev b 1	M	0.707560234
Taraxacum officinale	Tar o [Pollen]	E	0.700310884
Acarus siro	Aca s	E	0.698592024
Culicoides	Cul o 6	M	0.686143673
Bos domesticus	Bos d 5.0102	M	0.672595402
Platanus acerifolia	Pla a 8	M	0.669199521
Musa x paradisiaca	Mus xp	E	0.660557
Equus caballus	Equ c [Milk]	E	0.641591098
Cladosporium herbarum	Cla h	E	0.627312615
Triticum aestivum	Tri a [Pollen]	E	0.625247924
Canis familiaris	Can f 1	M	0.61595743
Saccharomyces cerevisiae	Sac c	E	0.600429374
Culicoides obsoletus	Cul nu 2	M	0.598849656
Carpinus betulus	Car b	E	0.571083305
Arachis hypogaea	Ara h 1	M	0.559353341
Alternaria alternata	Alt a 1	M	0.549001691
Leucanthemum vulgare	Leu vu	E	0.505030455
Brassica	Bra n	E	0.496039753
Acer saccharinum	Ace s	E	0.343599366
